# Mesenteric Cyst: A Case Report

**DOI:** 10.7759/cureus.34325

**Published:** 2023-01-29

**Authors:** Hanan E Alqurashi, Abdulrahman A Alaryni, Rani A Alsairafi, Ahlam M Alharbi, Abdullah A Alaqla

**Affiliations:** 1 General Surgery, Al-Noor Specialist Hospital, Makkah, SAU; 2 General Surgery, King Faisal Hospital, Makkah, SAU; 3 General Surgery, College of Medicine, Umm Al-Qura University, Makkah, SAU; 4 General Surgery, King Abdulaziz University, Makkah, SAU

**Keywords:** excision surgery, general surgery, cysts, mesentery, mesenteric cyst

## Abstract

Mesenteric cysts are rare benign abdominal lesions that possess the risk of malignant transformation in 3% of reported cases. Most cysts are asymptomatic and diagnosed incidentally or during the management of their complications. In the majority of cases, they arise from the mesentery of the small bowel, followed by the mesocolon. We present a case report of a 20-year-old female with an abdominal mesenteric cyst.

## Introduction

Mesenteric cysts are rare benign abdominal lesions that possess the risk of malignant transformation in 3% of reported cases [1-4]. They most commonly present with variable and nonspecific symptoms; 40% of cases are incidental findings during physical examinations or imaging procedures [2,3]. They present in the first decade of life [5] and possess a 1:1 male: female ratio [1,6].

Cysts can occur in any part of the mesentery but most frequently originate from the mesentery of the small bowel (ileum: 60%) and mesocolon (ascending colon: 24%) [7]. These formations have several classifications, including one based on histopathology features. The main treatment option is complete surgical excision. This can be accomplished by laparotomy or laparoscopy [3,4].

The etiology of mesenteric cysts is unclear, but a failure of the lymph nodes to communicate with the lymphatic or venous systems or the blockage of the lymphatic system as a result of previous pelvic surgery, trauma, pelvic inflammatory disease, infection, endometriosis, or neoplasia have been suggested as contributing factors [8,9]. 

## Case presentation

A young 20-year-old unmarried female consulted our hospital, King Faisal Hospital, for a painless abdominal swelling in the right upper quadrant, lasting three months, increasing gradually in size, and associated with nausea and anorexia.

There was no history of pain, vomiting, bowel habit changes, hematemesis, melena, or urinary symptoms. Additionally, there was no fever or history of abdominal trauma. She had a history of weight loss over the past several months associated with noticeable pallor but without a history of dizziness or palpitations. She had regular menstrual cycles; there was no family history of a similar mesenteric disease, malignancy, or any congenital anomalies. Her past medical history was unremarkable, with no previous history of surgery or medication use.

On clinical examination, she was conscious, oriented, and not pale. Her vital parameters were found to be within normal limits. On abdominal examination, there were no signs of abdominal distension or swelling, but a round mobile cystic mass was palpated in the right hypochondrial region, which was more prominent when she lay on her left side. It was not tender and about 7 × 10 cm in size; bowel sounds were positive. There were no skin changes or dilated veins. On digital rectal examination, no external pathologies were found; no masses were palpable. There were also no palpable lymph nodes.

**Figure 1:**
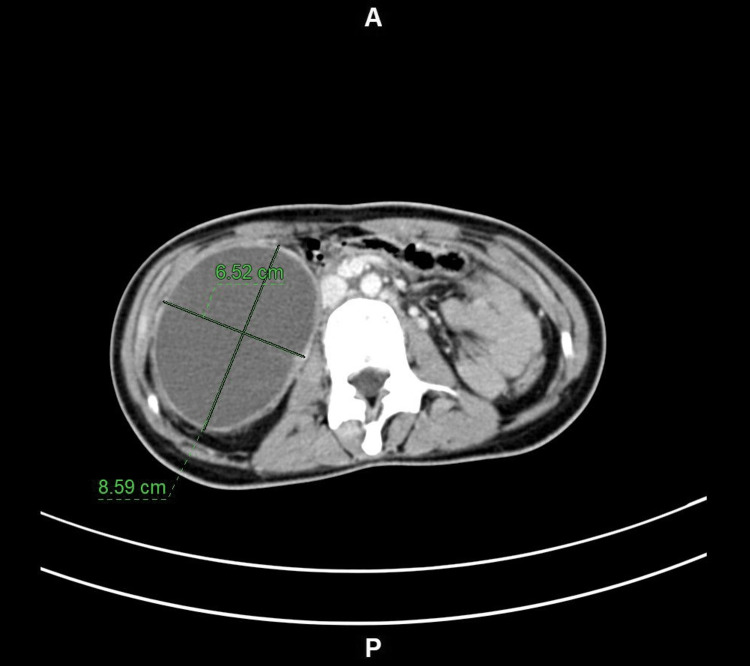
Computed tomography view of the mesenteric cyst

Blood tests were normal, with a hemoglobin of 12.4 g/dl. Liver function tests showed direct bilirubin of 0.02 mg/dl, total bilirubin of 0.23 mg/dl, alanine aminotransferase (ALT) of 11 IU/L, and aspartate aminotransferase (AST) of 12 IU/L. An abdominal CT scan showed a large well-defined cystic lesion (8.5 × 6.5 cm) with a peripherally enhancing wall seen in the right upper quadrant compressing the second part of the duodenum and exerting a mass effect on the adjacent large bowel loops, mainly at the hepatic flexure (Figure 1).

**Figure 2:**
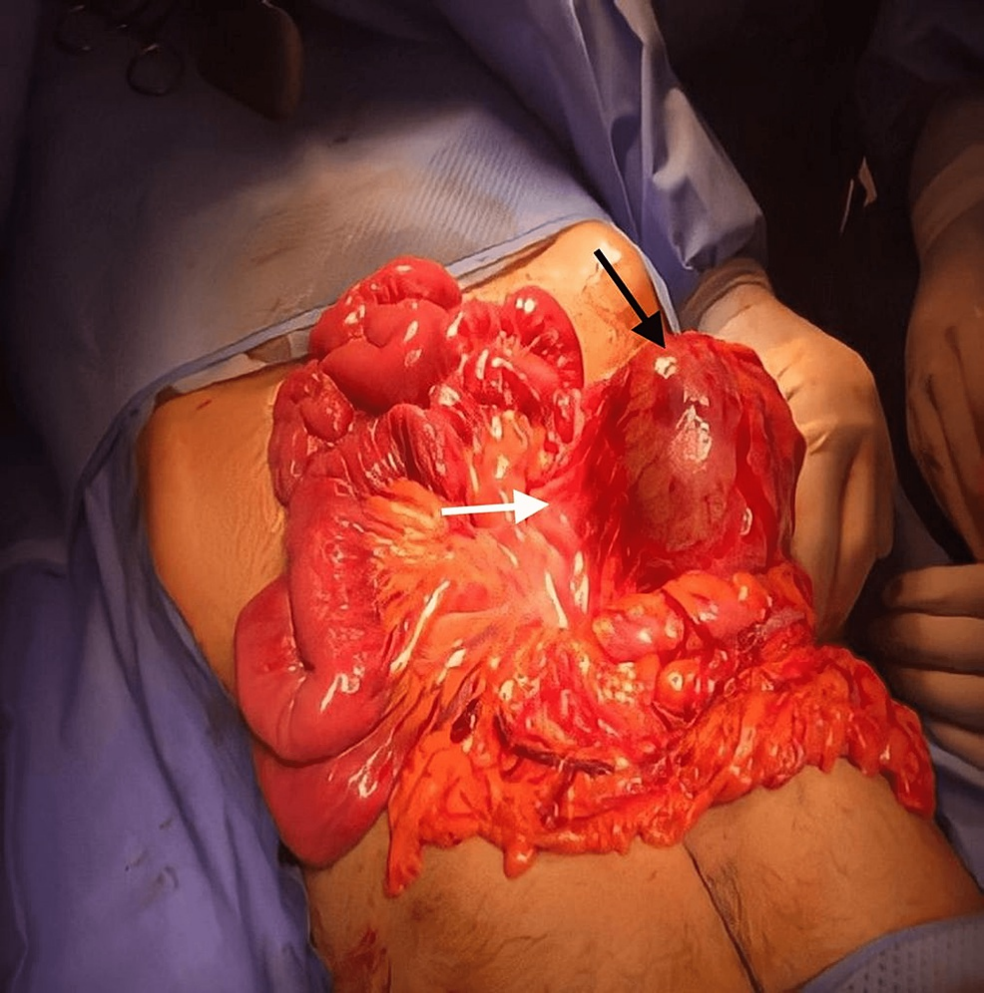
Intra-operative image showing a 9 x 9 cm cyst (black arrow) attached to and compressing parts of the duodenum and transverse colon mesentery (white arrow)

The patient was taken to an operative theatre for an exploratory laparotomy through a midline incision that revealed the following findings: a 9 x 9 cm cyst arising from transverse mesocolon and compressing parts of the duodenum. The cyst contained brownish fluid. Cautious dissection of the adhesions between the cyst and its surroundings was performed. Surgical resection of the cyst necessitated partial resection of about 8 cm of transverse colon with side-to-side anastomosis due to the adherent nature of the cyst to the colon and its mesentery (Figure 2).

The histological report revealed an intact cyst measuring 6.5 cm and separate flattened fatty tissue measuring 9 x 3 x 0.5 cm. The cyst contained brownish fluid, and a chocolate-like material was attached.

**Figure 3:**
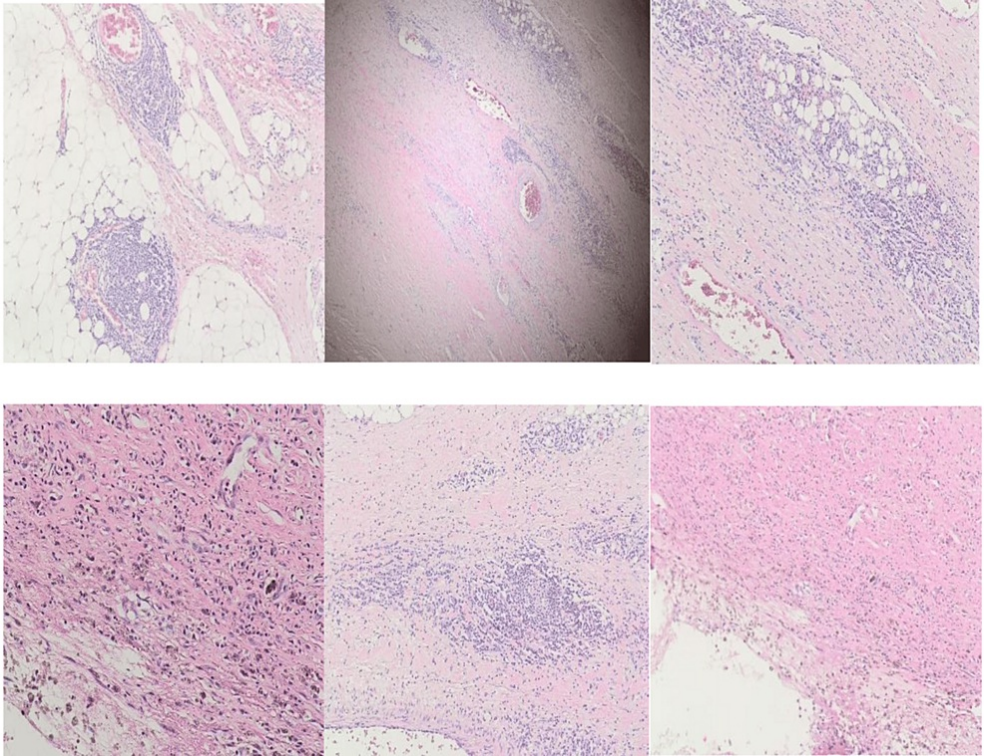
H&E-stained sections display a fibrous wall infiltrated by chronic inflammatory cells; lymphoid aggregates in the lumen show macrophages

H&E-stained sections of three representative paraffin blocks display a fibrous wall infiltrated by chronic inflammatory cells; lymphoid aggregates in the lumen show macrophages. No malignancy was present, and separate mature well-vascularized fatty tissue was also present (Figure 3). This confirmed the diagnosis of abdominal mesenteric cyst of the subtype pseudocyst.

The postoperative course was uneventful; she was fine throughout her hospital stay which lasted five days after surgery, and then was discharged home in good medical condition.

## Discussion

Most mesenteric cysts are benign lesions, but they tend to present with variable and nonspecific symptoms, such as abdominal pain, nausea, vomiting, anorexia, and a change in bowel habits; they can additionally present with complications, including intestinal obstruction, volvulus, torsion, bleeding, or rupture [2]. Cysts occur more often in the small (66%) than the large (33%) intestine. In the large bowel, they typically occur in the right colon but rarely in the mesentery of the descending colon, sigmoid colon, or rectum [10].

Diagnosis of mesenteric cysts can be challenging, as cysts mimic other pathologies, such as pancreatic pseudocysts or cystic tumors, pelvic diseases, and aortic aneurysms. A preoperative diagnosis can be achieved using imaging techniques (e.g., ultrasonography (US), CT, nuclear MRI) [8-9,11-13]. CT scan is essential to localize the cystic mass and the anatomical structures involved; it also helps in adequately planning the surgical approach.

The etiology of mesenteric cysts is unclear, but a failure of the lymph nodes to communicate with the lymphatic or venous systems or the blockage of the lymphatic system as a result of previous pelvic surgery, trauma, pelvic inflammatory disease, infection, endometriosis, or neoplasia have been suggested as contributing factors [8,9]. 

Complete surgical excision is the first-choice therapy to avoid recurrence and the possible risk of malignant transformation and may require the removal of part of the mesentery with the mass. The removal of the cyst can be achieved via laparotomy or laparoscopy [8].

The decision of surgery type depends on the dimensions of the cyst, its relationship to the major abdominal structures, as well as the surgeon’s experience [14].

In this case, laparotomy was the procedure of choice. The patient underwent complete surgical resection of the cyst, necessitating partial colon resection due to the adherence of the cyst to the colon and its mesentery.

## Conclusions

The results of the patient's preoperative diagnostic examination strongly suggested the presence of a mesenteric cyst; intraoperatively, the position and size of the cyst corresponded to the preoperative radiographic findings. The histological examination displayed a benign cyst with a fibrous wall infiltrated by inflammatory cells and lymphoid aggregates. The lumen shows macrophages with no malignant cells. Thus, complete resection of the lesion with partial resection of the colon was performed. The treatment of choice for mesenteric cysts is complete surgical excision. 
